# The effect of TLR4 on the growth and local inflammatory microenvironment of HPV-related cervical cancer in vivo

**DOI:** 10.1186/s13027-020-0279-9

**Published:** 2020-02-17

**Authors:** Ninghong Jiang, Feng Xie, Limei Chen, Fang Chen, Long Sui

**Affiliations:** 1 Medical center for diagnosis and treatment of cervical disease, Obstetrics and Gynecology Hospital, Fudan University, Shanghai, 200011 China; 2 Shanghai Key Laboratory of Female Reproductive Endocrine Related Diseases, Shanghai, 200011 China

**Keywords:** HPV, Cervical cancer, Local inflammatory microenvironment

## Abstract

**Background:**

Cervical cancer is the most common malignancy of the female lower genital tract. In our previous study, we found that TLR4 promotes cervical cancer cell growth in vitro. The aim of this study was to further explore the role of TLR4 in HPV-related cervical cancer in vivo by using a nude mouse xenograft model.

**Methods:**

Cervical cancer-derived HeLa and CaSki cells (5 × 10^7^/mL) were either stimulated with an optimal concentration of LPS for the appropriate time (HeLa cells were treated with 1 μg/mL LPS for 1 h, and CaSki cells were treated with 2 μg/mL LPS for 1.5 h) or transfected with TLR4 shRNA and then injected subcutaneously into the dorsal right posterior side of nude mice. The shortest width and longest diameter of the transplanted tumors in the nude mice were measured every 3 days.TLR4, IL-6,iNOS, IL-8,COX-2, MIP-3α, TGF-β1 and VEGF expression levels in the transplanted tumor tissue were detected by immunohistochemistry.

**Results:**

The tumor formation rate was 100% in both HeLa and CaSki nude mouse groups. The tumors grew faster, and the cachexia symptoms were more serious in the LPS groups than in the control group. In contrast, the tumors grew slower, and the cachexia symptoms were milder in the TLR4-silenced groups. TLR4, iNOS, IL-6, MIP-3α and VEGF were highly expressed in the transplanted tumor tissues from the LPS groups, and their expression levels were decreased in the TLR4-silenced groups.

**Conclusion:**

TLR4 expression is closely associated with the tumorigenesis and growth of HPV-positive cervical cancer; TLR4 promotes HPV-positive cervical tumor growth and facilitates the formation of a local immunosuppressive microenvironment. Eventually, these conditions may lead to cervical cancer development.

## Background

Cervical cancer is the fourth most common tumor among women in the world, with over 500,000 new cases in 2018 [1]. At present, many studies have noted that persistent inflammatory conditions play a crucial role in tumorigenesis, such as in hepatocellular carcinoma [2, 3]. Chronic inflammation promotes the malignant transformation of the epithelium by stimulating free radical production and suppressing the immune system. Furthermore, researchers found that nitrative and oxidative DNA damage induced by chronic inflammation promoted genomic abnormalities caused by HPV oncoproteins, which play a vital role in cervical carcinogenesis [4].

Human papillomavirus (HPV) is the most common sexually transmitted virus, and infection is closely associated with cervical cancer, causing significant morbidity and mortality [5]. High-risk types of HPV, especially HPV18 and HPV16, promote the tumorigenesis and progression of the majority of cervical cancers [6]. Multiple processes and signaling pathways are altered by HR-HPV E6 and E7 oncoproteins in cervical carcinogenesis, including the inactivation of two important tumor suppressor pathways (pRB and p53). Researchers have identified that E6 can induce p53 degradation by binding to the ubiquitin ligase E6AP, inhibit p53-dependent signaling upon stress stimuli, and contribute to tumorigenesis. In addition, the E7 oncoprotein associates with the retinoblastoma family of proteins (pRb, p107 and p130) and interrupts their connection with the E2F family of transcription factors, thereby transactivating the cellular proteins required for cellular and viral DNA replication. Additionally, E6 and E7 can activate the PI3K/Akt pathway to increase cell proliferation [7, 8]. E6 degrades PDZ-containing proteins by targeting p53 and then activates the Wnt/β-catenin pathway [9, 10]. Moreover, telomerase activation is critical for the immortalization of primary human keratinocytes by high-risk HPV E6, and E6 can increase telomerase activity by upregulating telomerase reverse transcriptase (TERT) [11]. Besides, E6 and E7 can deregulate cellular microRNA expression, which results in cellular signaling pathway alterations [12]. Thus, the coordinated interaction of various pathways involving proteins and other biomolecules contributes to HPV-related cervical carcinogenesis.

Toll-like receptors (TLRs), a type of pattern recognition receptor (PRR), recognize endogenous and exogenous pathogens to activate innate and adaptive immune responses [13]. Some studies have suggested that TLRs function in various types of carcinomas by inducing inflammation. Notably, studies on drugs that target TLR signaling pathways have been conducted to test the effect of pesticides on inflammation and cancers [14]. TLR4, one of numerous TLRs, can be stimulated by exogenous pathogens, such as bacterial lipopolysaccharide (LPS), and then respond to the infection by regulating the immune system. OnceTLR4 is activated, mitogen-associated protein kinases (MAPKs) and nuclear factor (NF)-κB are stimulated, which are signaling pathways downstream of TLR4, and genes encoding inflammatory cytokines are then upregulated. Previous studies have shown that TLR4 plays a vital role in cancer development [15–17]. For instance, researchers have suggested that the TLR4 pathway accelerates colon cancer cell migration [15] and protects cancer cells against apoptosis and immune surveillance [13]. Moreover, it has been documented that prostate cancer cells are significantly inhibited in terms of tumorigenicity, survival and invasion if TLR4 is down-regulated, while colon cancer death is accelerated [18, 19], which illustrates that TLR4 plays an important role in carcinogenesis.

However, the relationship between TLR4 and cervical cancer development remains controversial, and the carcinogenic mechanism has not been fully elucidated. In our previous study, we explored the relationship between TLR4 and cervical cancer cells in vitro and found that TLR4 promoted proliferation and apoptosis resistance in HPV-related cervical cancer cells [20]. Moreover, proinflammatory cytokines are highly expressed in HPV-related cervical cancer cells when the TLR4/MyD88/NF-κB pathway is activated, which indicates that TLR4 promotes cervical cancer progression via the formation of an immunosuppressive microenvironment [20]. In this study, a nude mouse xenograft model was used to further investigate the role of TLR4 in HPV-related cervical cancer in vivo to verify TLR4 function in a multidimensional manner. Our results indicated that TLR4 expression is closely related to HPV-related cervical cancer; furthermore, TLR4 promoted the growth of cervical tumors and facilitated the formation of a local immune microenvironment both in vitro and in vivo, thus promoting the development and progression of cervical cancer. This study further provides evidence for the clinical treatment of cervical cancer by targeting TLR4-relevant molecules.

## Methods

### Cervical Cancer cell lines and cell culture

HeLa and CaSki are common human cervical cancer cell lines with different HPV types. The HeLa cell line has the HPV 18 genome integrated into the human genome, and the identified HPV 18 integration sites thus far include 8q24, 9q31–q34, 5p11–15, 9q31–34 and 22q12–13.In the CaSki cell line, 2p23, 8q13, 8q24.3, 10q26, 11q13, 11q23–q25, 14q11.2, 19p13.1 and 20p11.2 are the sites where the HPV 16 genome is integrated into the human genome. Moreover, HPV type 18 transcripts in the HeLa cell line reside at 8q24, while HPV type 16 transcripts in the CaSki cell lines reside in the 13q21–22 chromosome region [21]. For this study, the HeLa and CaSki cells were purchased from the Chinese Cell Bank (Shanghai, China). The cells were cultured in DMEM/F12 medium (HyClone, Logan, UT, USA) with 10% fetal bovine serum (FBS) (Gibco, Grand Island, NY, USA). Both CaSki and HeLa cells were maintained in 5% CO_2_ at 37 °C, and cell passaging was performed every 3 days using trypsin.

### shRNA transfection

To efficiently suppress TLR4 expression, a small short hairpin RNA (shRNA) specifically targeting TLR4 was synthesized by GeneChem Co., Ltd. (Shanghai, China).TheTLR4 target sequence was AAGTAGTCTAGCTTTCTTA, which was shown to have a silencing effect in our previous study. In our previous study, we found that TLR4-shRNA-3 (TLR4–3) had the greatest TLR4 silencing efficiency compared with 2 other shRNAs (TLR4–1 and TLR4–2);therefore, we used TLR4–3 in this study [20]. Nontargeting shRNA served as a negative control and was also designed by GeneChem Co., Ltd., Shanghai, China).CaSki and HeLa cells were trypsinized using 0.25% trypsin in cell culture medium on the day before transfection. Then, the trypsinized cells were seeded in 6-well plates at a density of 4 × 10^5^ cells per well. When the cells reached 70% confluence, a Lipofectamine 3000 kit (Invitrogen, Carlsbad, CA, USA) was used to transfect the cells according to the manufacturer’s instructions. Three groups were included in this experiment: a TLR4-knockdown group transfected with TLR4-shRNA; a negative control (NC) group transfected with NC-shRNA; and an untransfected control group. After 24 h, the cells were collected for subcutaneous injection in the dorsal right posterior side of the nude mice.

### Construction of the nude mouse Xenograft model

In a previous study, we found that TLR4 expression was higher in HeLa (HPV18+) and CaSki (HPV16+) cells than in C33A (HPV-) cells [20]. This positive correlation between TLR4 and HPV-related cervical cancer suggests that TLR4 may be involved in cervical cancer development and progression. To further confirm the crucial role of TLR4 in HPV-positive cervical cancer, a nude mouse xenograft model was used. For this study, 4-week-old female nude mice were purchased from Shanghai Slac Laboratory Animal Co., Ltd.,(Shanghai,China) and allowed to adapt to the surrounding environment for a week before the experiment. Then, the mice were divided randomly into groups (*n* = 4 for each group) as follows: LPS stimulation group: 5 × 10^7^/mL HeLa and CaSki cells were treated with an optimal concentration of LPS for the appropriate time (HeLa cells were treated with 1 μg/mL LPS for 1 h, and CaSki cells were treated with 2 μg/mL LPS for 1.5 h); the optimal concentration of LPS was determined in our previous study [20]. Then, these treated cells were injected subcutaneously into the dorsal right posterior side of the nude mice**.** Multipoint injections in the tumor growth area were performed every 2 days with200 μLof LPS at the optimum concentration. TLR4 shRNA-transfected group: 5 × 10^7^/mL HeLa and CaSki cells were transfected with TLR4 shRNA in vitro and then injected subcutaneously into the dorsal right posterior side of the nude mice after24 h. Multipoint injections in the tumor growth area were performed every 2 days with 200 μL of cell culture medium containing plasmids and transfection reagents. The NC group and control group were injected with 200 μL of cell culture medium containing an empty vector and transfection reagents or medium containing only transfection reagents, respectively. Then, the longest diameter and shortest width of the transplanted tumors in the nude mice were measured every 3 days.

### Immunohistochemistry

Tumor tissue of the nude mice was fixed in 10% formalin buffer for 1 day, and paraffin was used to embed the tissues; then, the fixed tissue samples were sectioned. After deparaffinization and gradient hydration, antigen retrieval was performed. Then, the sections were incubated with polyclonal anti-TLR4,COX-2,iNOS,IL-6,IL-8, MIP-3α, TGF-β1 and VEGF antibodies(1:500, Abcam, Cambridge, MA, UK) and placed in a 4°Crefrigerator overnight. The following morning, horseradish peroxidase (HRP)-conjugated anti-TLR4, COX-2,iNOS,IL-6,IL-8, MIP-3α, TGF-β1 and VEGF secondary antibodies (1:200; Arigobio, Taiwan, ROC) were used. Then, the sections were incubated in 3,3′-diaminobenzidine (DAB, Beyotime, Shanghai, China). Following counterstaining with hematoxylin, the sections were washed and dehydrated, and a cover slip was mounted with neutral balsam. Image-Pro Plus software (IPP; produced by Media Cybernetics Corporation, USA) 6.0 was used for the quantitative analysis of the IHC results.

### Statistical analysis

The data are expressed as the mean ± SD. GraphPad Prism 6.0 software (San Diego, CA, USA) was used for the statistical analysis. All the data were analyzed by t-tests, and *p* values less than 0.05 were considered statistically significant.

## Results

### TLR4 was highly expressed in transplanted tumor tissue from the LPS-stimulated group and downregulated in that from the TLR4-silenced group

**Fig. 1:**
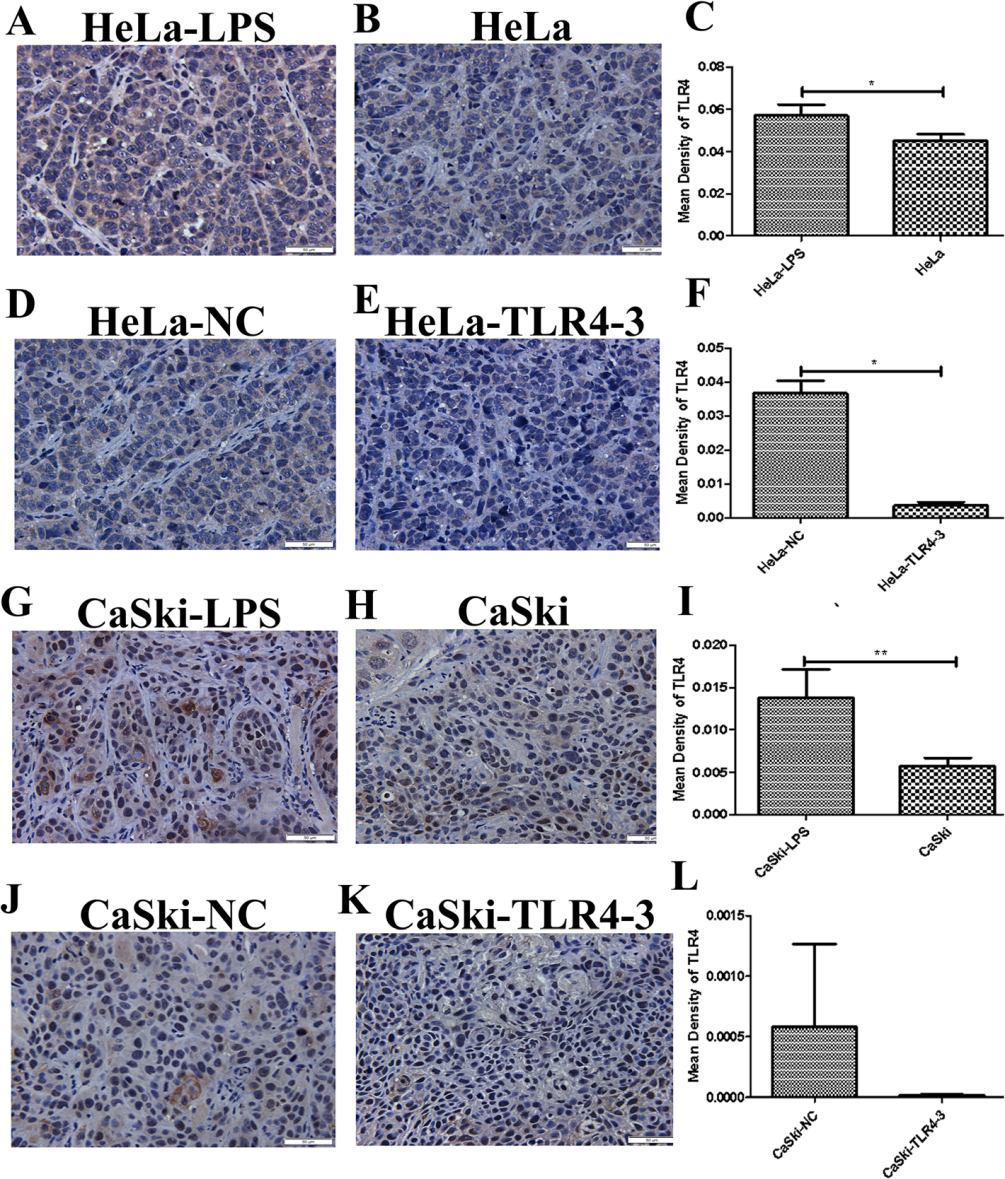
TLR4 immunohistochemical (IHC) analysis (× 400) of transplanted tumor tissue from a nude mouse xenograft model. The TLR4–3 group was the TLR4-knockdown group transfected with TLR4-shRNA; the NC group was the negative control group transfected with NC-shRNA. (**c** ,**f** ,**i** ,**l**) Quantitative analysis of TLR4 expression in transplanted tumor tissues with different treatments

Because the purpose of this study was to observe the effects of different TLR4 levels on the growth and other indexes of xenograft tumors, the first step was to determine whether certain experimental treatments could alter TLR4 levels in the transplanted tumor tissues to ensure the validity of the subsequent experiments. Here, TLR4 expression in the transplanted tumor tissues was analyzed by immunohistochemistry. The results showed that TLR4 was highly expressed in the LPS-stimulated HeLa and CaSki groups but downregulated in the TLR4-silenced HeLa and CaSki groups (Fig. 1).

### TLR4 promotes the growth of tumors transplanted in nude mice

**Fig. 2:**
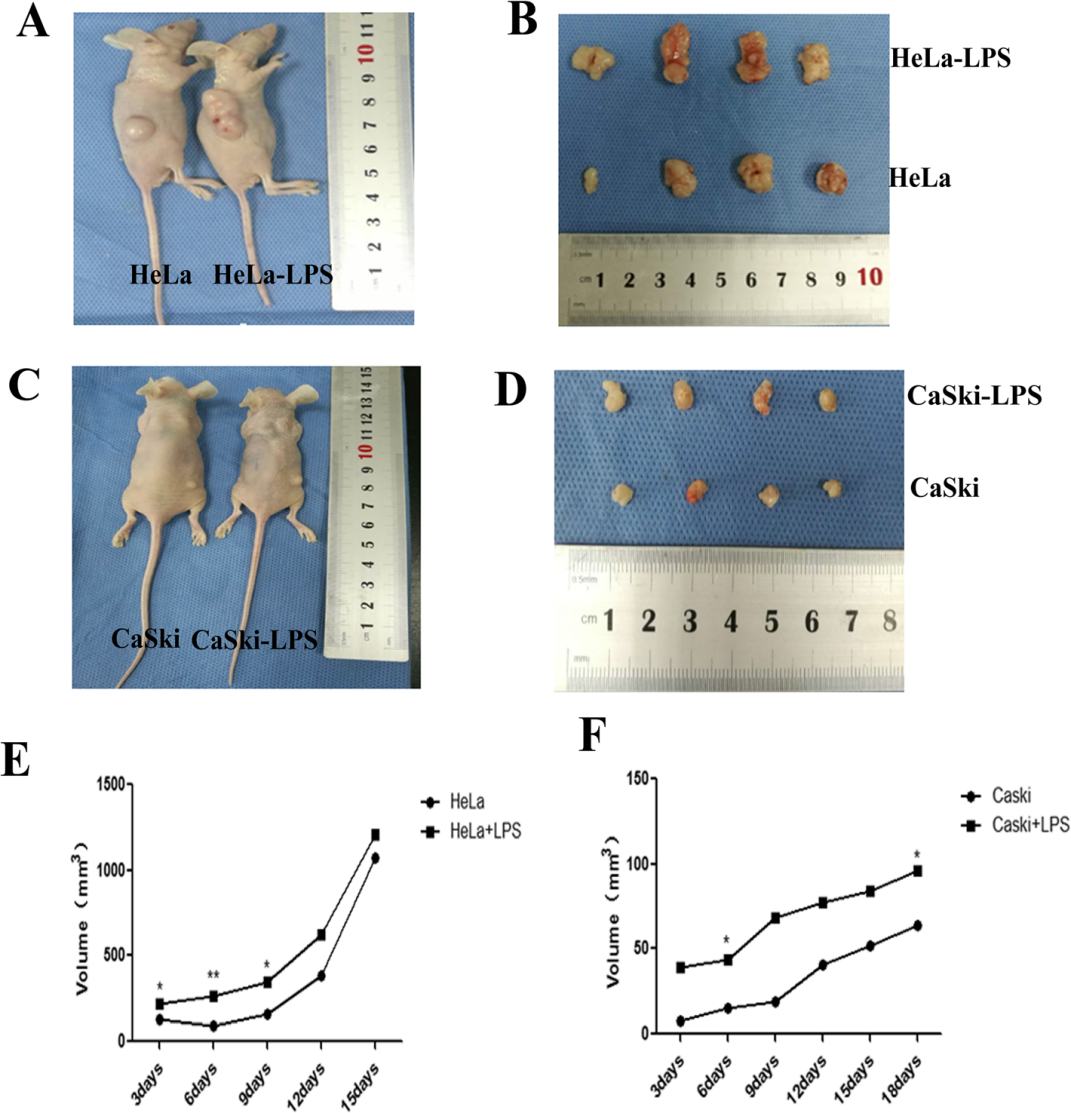
(**a**-**d**) Tumor sizes of the nude mouse xenograft model with LPS treatment. **e**, **f** Tumor growth chart for the nude mouse xenograft model

**Fig. 3:**
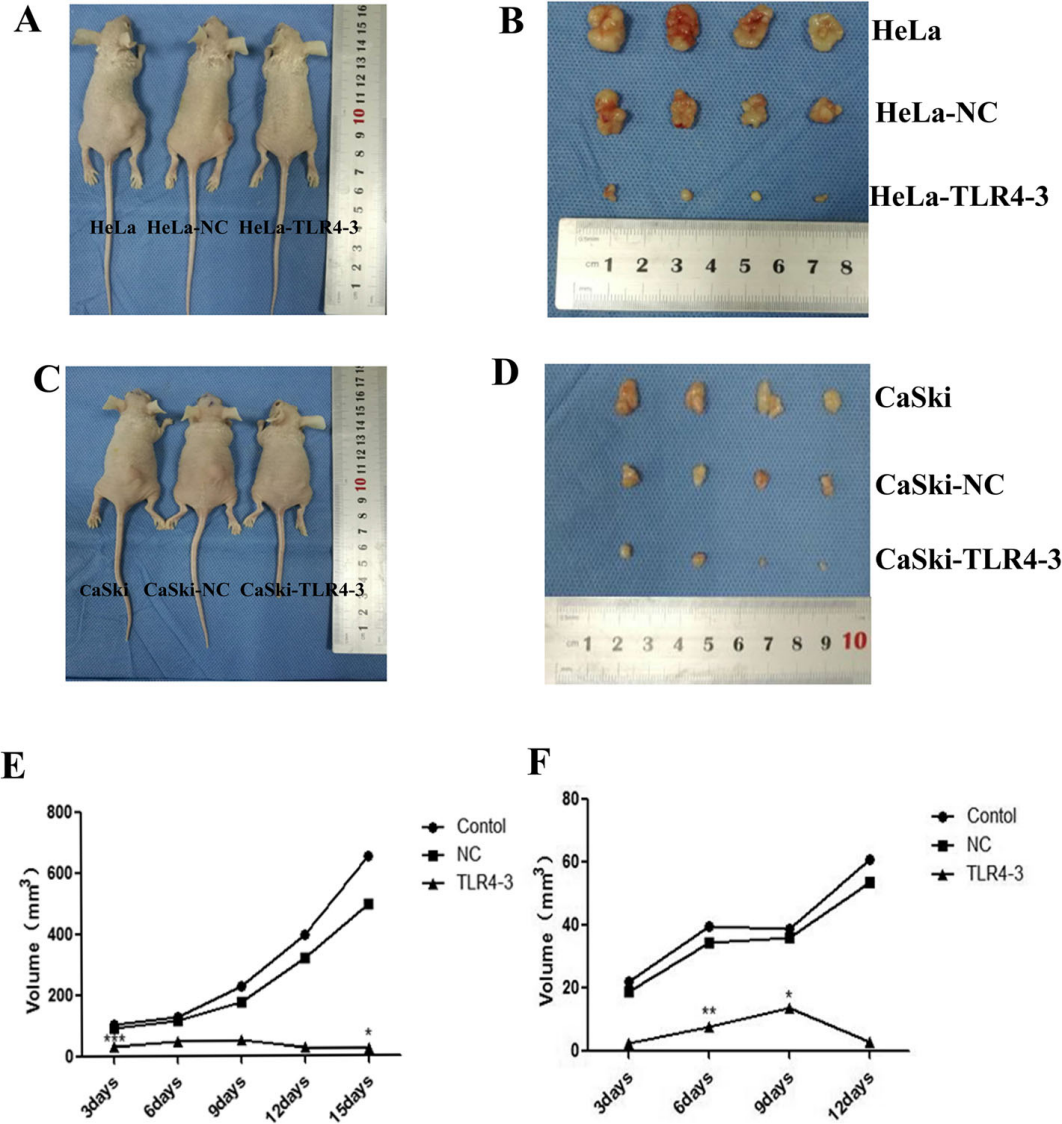
(**a**-**d**) Tumor sizes of the nude mouse xenograft model with TLR4 silencing. **e**, **f** Tumor growth chart for the nude mouse xenograft model

When nude mice were injected on the dorsal right posterior side with HeLa and CaSki cells subjected to different treatments, tumors gradually formed; the tumor formation rate was 100%. Then, as time increased, the tumor volume increased gradually. In addition, the physical condition of the nude mice became worse with the enlargement of the tumors. The amount of subcutaneous fat decreased, and the nude mice became increasingly tired and listless. Furthermore, the decrease in food intake became increasingly serious. As shown in Fig. 2, the volume of the tumors was larger in the LPS-stimulated group than in the control group, and the amount of subcutaneous fat was much lower. The tumors were nodular and not easily separated from the skin, and there were areas of hemorrhaging and necrosis on the tumor surface (Fig. 2 b, d). However, in the TLR4-silenced group, the opposite results were observed (Fig. 3).

### TLR4 facilitates the formation of a local inflammatory microenvironment in nude mouse transplanted tumors

**Fig. 4:**
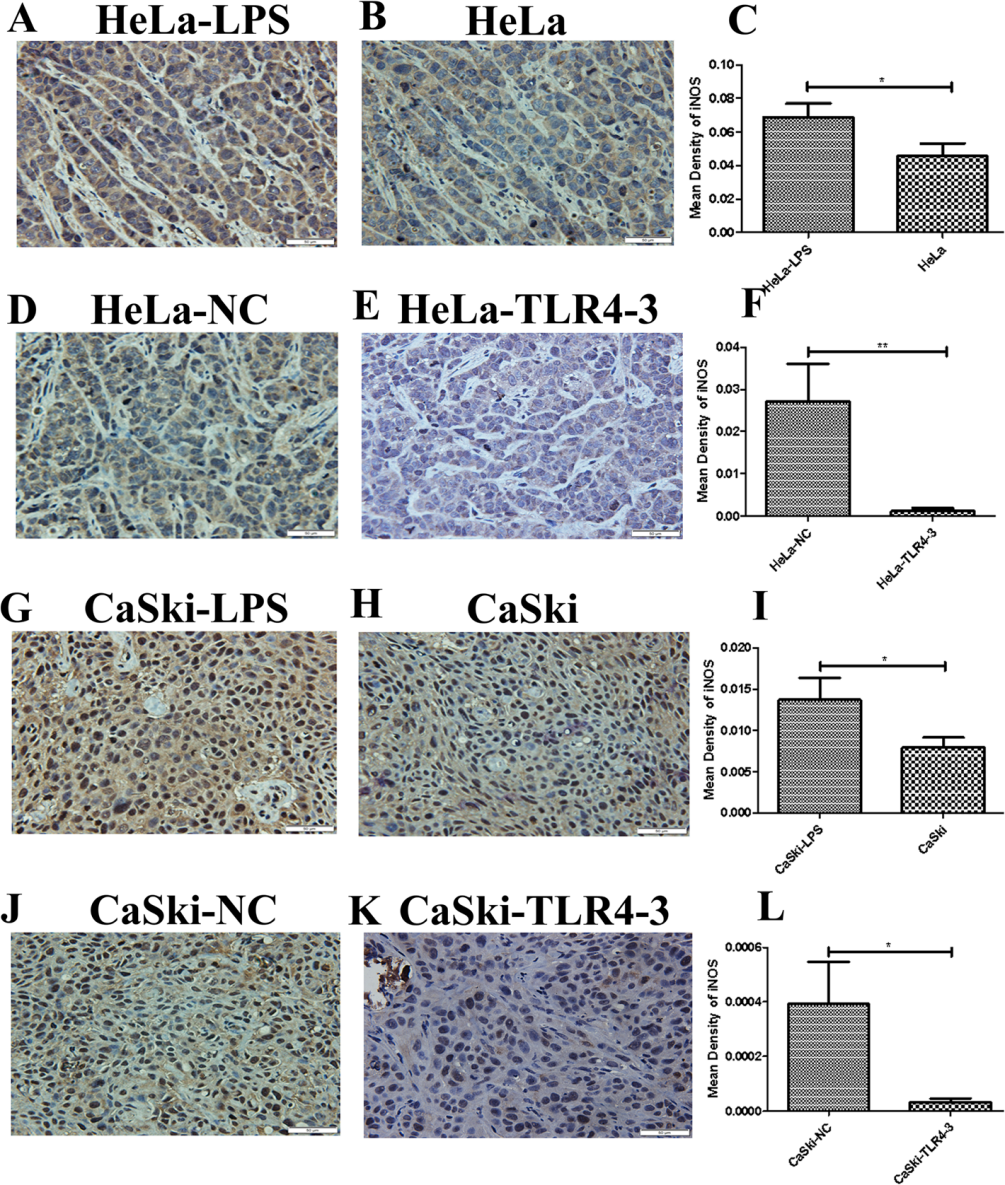
iNOS immunohistochemical (IHC) analysis (× 400) of transplanted tumor tissue from the nude mouse xenograft model. (**c** ,**f** ,**i** ,**l**) Quantitative analysis of iNOS expression in transplanted tumor tissues with different treatments

**Fig. 5:**
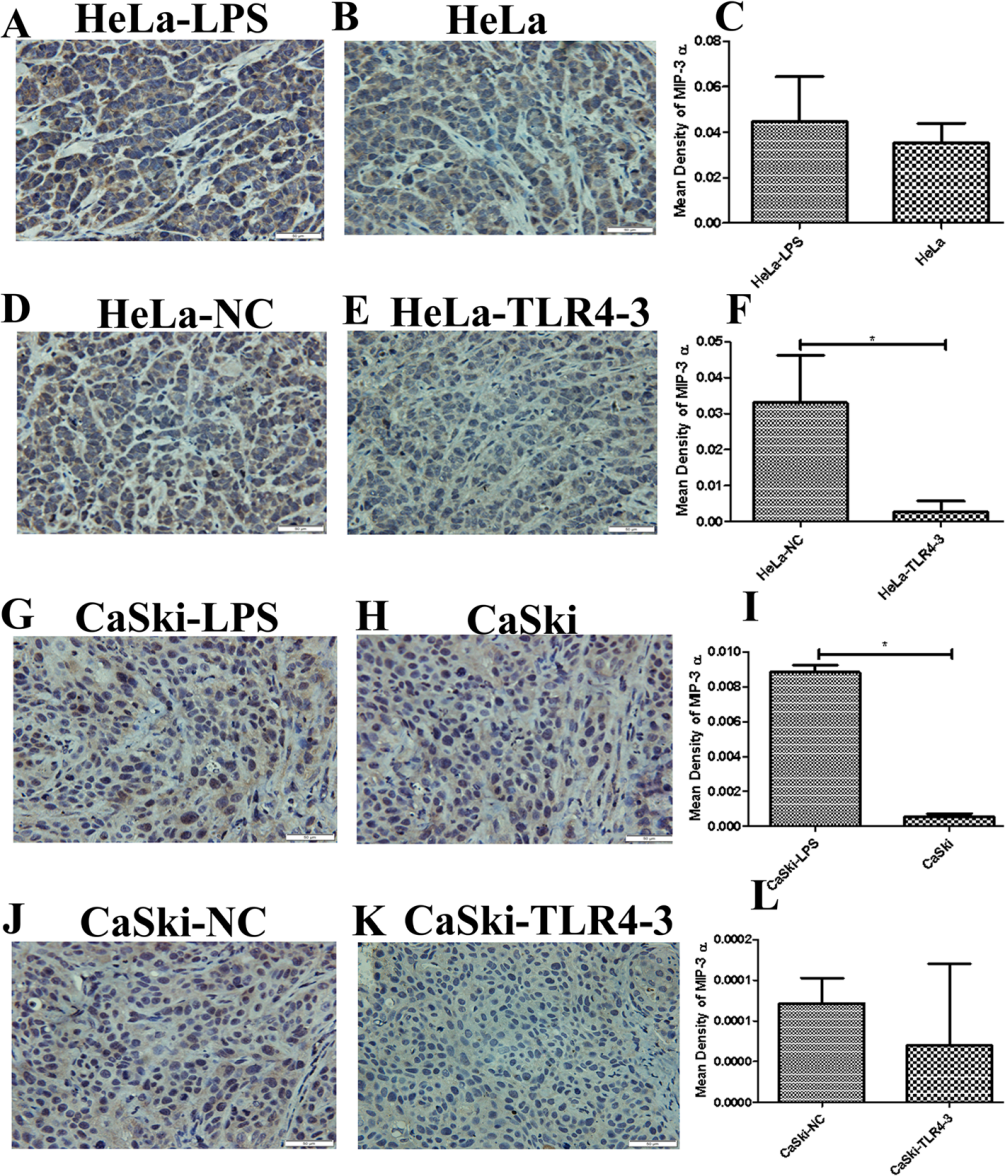
MIP-3α immunohistochemical (IHC) analysis (× 400) of transplanted tumor tissue from the nude mouse xenograft model. (**c**, **f**, **i**, **l**) Quantitative analysis of MIP-3α expression in transplanted tumor tissues with different treatments

**Fig. 6:**
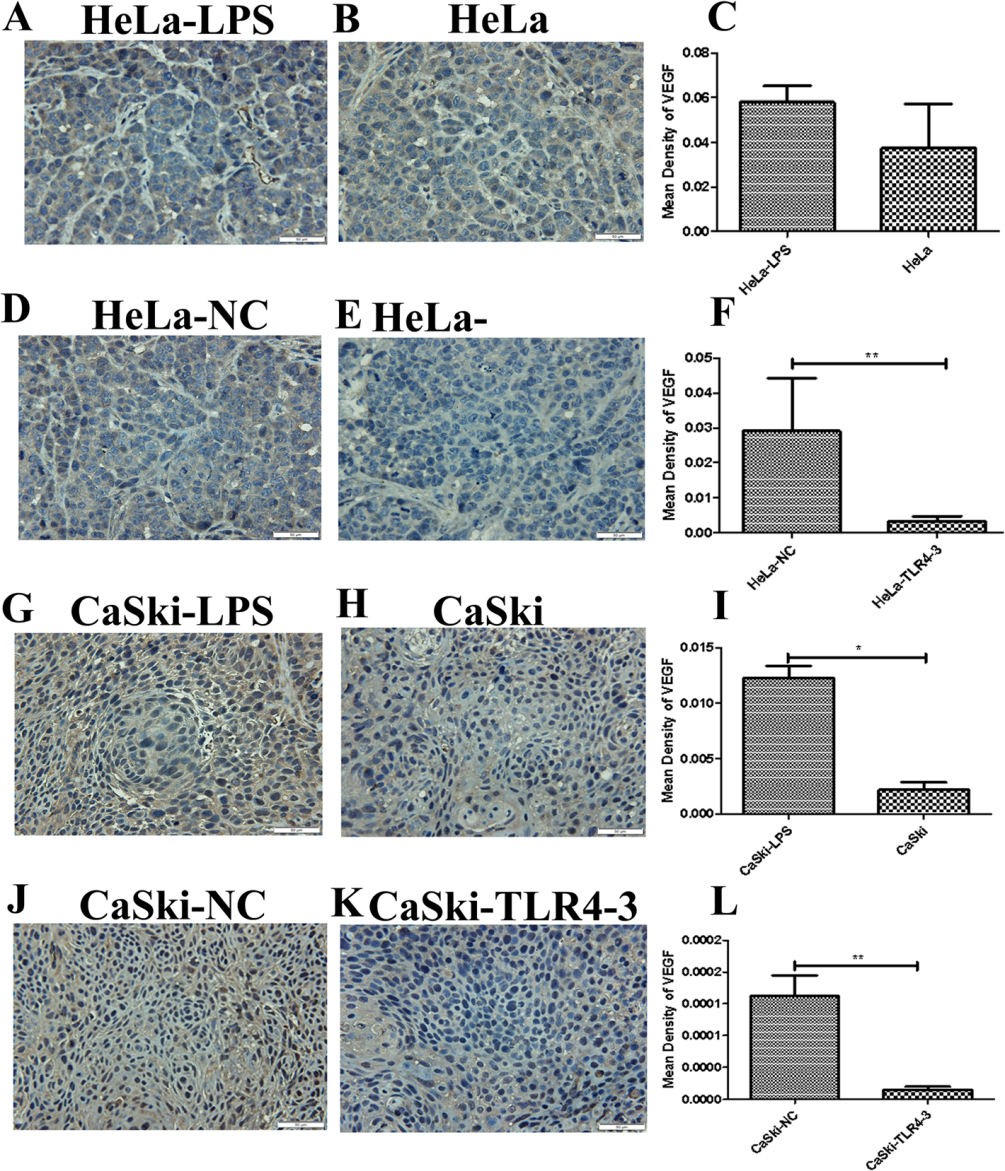
VEGF immunohistochemical (IHC) analysis (× 400) of transplanted tumor tissue from the nude mouse xenograft model. (**c**,**f**,**i**,**l**) Quantitative analysis of VEGF expression in transplanted tumor tissues with different treatments

**Fig. 7:**
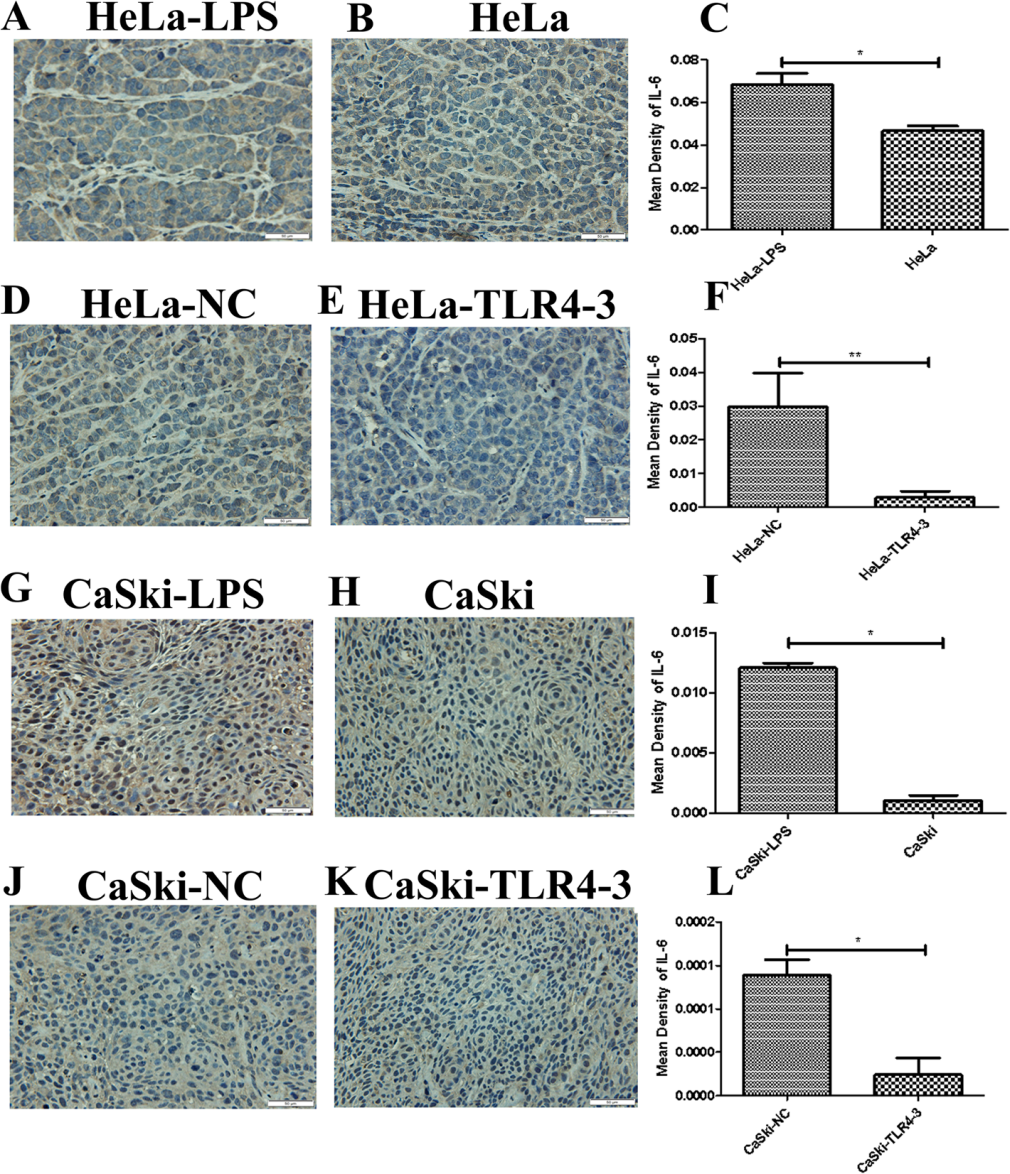
IL-6 immunohistochemical (IHC) analysis (× 400) of transplanted tumor tissue from the nude mouse xenograft model. (**c** ,**f** ,**i** ,**l**) Quantitative analysis of IL-6 expression in transplanted tumor tissues with different treatments

It has been reported that the inflammatory microenvironment plays a key role in the development of solid tumors. However, whether TLR4 can promote the HPV-related cervical cancer inflammatory microenvironment in vivo remains to be explored. In this study, immunohistochemistry was used to analyze the expression of inflammatory factors, such as COX-2, iNOS, IL-6,IL-8,MIP-3α,TGF-β1 and VEGF, in the transplanted tumor tissues. We found that iNOS, MIP-3α,VEGF and IL-6 were highly expressed in the LPS groups**.** No significant changes were found in the expression levels of COX-2, IL-8 and TGF-β1 (results not shown). In contrast, the expression levels of iNOS, MIP-3α, VEGF and IL-6 were decreased in the TLR4-silenced groups (Figs.4, 5, 6 and 7**)**,and the levels of COX-2, IL-8 and TGF-β1 were not significantly changed (results not shown).

## Discussion

Studies have shown that chronic inflammation is a stimulatory factor in many cancers [18]. The mechanism of chronic inflammation leading to tumorigenesis and cancer development has not been clearly elucidated. Some experts have noted that tumorigenesis and cancer development may be associated with the effects of inflammation. For example, IL-6, a common inflammatory cytokine that can activate reactive oxygen species and then induce genetic mutations, is produced during the chronic inflammation process. In addition, other studies have suggested that DNA damage and tumor-initiating mutations that cause tumorigenesis, apoptosis resistance, proliferation, migration, invasion and metastasis can also be induced by chronic inflammation [22–24]. In addition, Shimazaki J, etal [25]. noted that the expression level of IL-6 is positively correlated with colon cancer development.

TLR4, a type of pathogen recognition receptor, is a key sensor that recognizes viruses such as HPV and subsequently induces a strong immune response. TLR4, which is detected not only in immune cells but also in numerous cancers, has been verified as an important medium that links inflammation and tumorigenesis [26–28]. A study showed that TLR4 promotes tumor metastasis and is closely related to poor prognosis in breast cancer [28]. Another study that analyzed 116 tissue samples from patients with different stages of colorectal disease found that patients with higher TLR4 tumor expression levels had a greater risk of disease progression and relapsed earlier than those with lower expression levels [29]. Moreover, in colitis-associated tumors, TLR4 has been shown to induce an inflammatory microenvironment [30]. Notably, the specific anatomic location of the cervix and sexual activity increase the susceptibility of the cervix to microbial infection, which can cause chronic inflammation in the cervix. Moreover, in our previous study, we found that TLR4 was highly expressed in cervical cancer and that TLR4 promoted proliferation and resistance to apoptosis in HPV-related cervical cancer cells. Furthermore, we found that TLR4 promoted the expression of proinflammatory cytokines such as COX-2, iNOS, IL-6, IL-8, MIP-3α, TGF-β1 and VEGF in HPV-related cervical cancer cells in vivo [20]. These results suggest thatTLR4 plays a significant modulatory role in cervical cancer pathogenesis and immunity.

To further confirm the role of TLR4 in HPV-related cervical cancer in vivo, a nude mouse xenograft model was used in this study. Additionally, two typical HPV-positive cervical cancer cell lines, HeLa and CaSki, were injected subcutaneously into the dorsal right posterior side of the nude mice. Then, the direct effect of TLR4 on tumor growth was observed carefully. Our results demonstrated that the tumorformation rate was 100% in both the HeLa and CaSki groups. Moreover, the tumors grew faster, and the cachexia symptoms were more serious when TLR4 levels were increased; the opposite effect on tumors was observed when TLR4 expression was downregulated. Furthermore, immunohistochemistry showed that iNOS, IL-6, MIP-3*α* and VEGF expression was positively correlated with TLR4 levels in transplanted tumor tissue in a TLR4-dependent manner.

iNOS, a common type of induciblenitric oxide synthase, can promote tumor development by facilitating genetic mutation and stimulating angiogenesis, metastasis, proliferation, and immunosuppression [31]. MIP-3α can not only create an inflammatory microenvironment but also recruit certain types of immunosuppressive cells, such as Treg, Th17, and Th22 cells, all of which accelerate cancer development and progression [32–35].VEGF is another important factor that can promote angiogenesis and lymphangiogenesis, which are vital processes in cancer development and progression, and it promotes tumor growth, metastasis and invasion [36]. In this study, our data suggested that TLR4 expression is closely correlated with HPV-positive cervical cancer. TLR4 promoted the growth of cervical tumors and facilitated the formation of a local immune microenvironment both in vitro and in vivo and eventually promoted the development and progression of cervical cancer.

## Conclusion

In conclusion, this study further confirms the hypothesis that TLR4 plays an important tumor-promoting role in HPV-related cervical cancer, which depends mainly on the formation of an immunosuppressive microenvironment. In addition, this study increases our understanding of the pathogenesis of HPV-related cervical cancer and provides clues to enable the exploration of new therapeutic methods to treat cervical cancer by targeting TLR4-relevant molecules.

## Data Availability

All data are available from the corresponding author.

## Abbreviations

COX-2: Cyclooxygenase-2
HPV: Human papillomavirus
IL-6: Interleukin-6
IL-8: Interleukin-8
iNOS: Inducible nitric oxide synthase
LPS: Lipopolysaccharide
MIP-3α: Macrophage inflammatory protein-3α
PRR: Pattern recognition receptor
shRNA: Short hairpin ribonucleic acid
TGF-β1: Transforming growth factor-β1
TLR4: Toll-like receptor 4
TLRs: Toll-like receptors
Treg: Regulatory T cell
VEGF: Vascular endothelial growth factor
